# A numerical assignment on local pressure capacity of the concrete under the head of straight anchor bolts

**DOI:** 10.1038/s41598-025-33348-1

**Published:** 2026-01-16

**Authors:** Sabry Fayed, Mohamed Ghalla, Abdulaziz Alaskar, Saad A. Yehia, Mohammed K. Alkharisi, EL-Said A. Bayoumi

**Affiliations:** 1 Civil Engineering Dept, Faculty of Engineering, Kafrelsheikh University, Kafrelsheikh, Egypt; 2 Department of Civil Engineering, College of Engineering, King Saud University, Riyadh, 11421 Saudi Arabia; 3 Civil Engineering Department, Higher Institute of Engineering and Technology, Kafrelsheikh, Egypt; 4 Department of Civil Engineering, College of Engineering, Qassim University, 52571 Buraidah, KSA Saudi Arabia; 5 Engineering Expert, Ministry of justice, Cairo, Egypt

**Keywords:** Local pressure, Concrete, Headed bars, Reinforcement, Failure, Pentagonal, Diameter, Cover thickness, Head shape, FEM, Engineering, Materials science

## Abstract

High-strength straight anchor bolts materials require longer embedment lengths in the concrete so the headed bars are employed to significantly reduce the required embedment length of these bars. But they impose concentrated stress on a small concrete surface under the head. In this study, behavior of headed bolts was numerically studied using 3D finite element method (FEM). First, an experimental program consists of four concrete specimens with variations only in head diameter was conducted. Second, FEM program consists of 22 concrete specimens was conducted. The effect of concrete cover thickness around the head was ranging from 85 mm to 510 mm. This variation resulted in a broad range of concrete cover-to-stud diameter ratios from 5 to 30. Additionally, studying the impact of head geometry (hexagonal, square, circular, and pentagonal) was performed. The effect of head diameter from 12 mm up to 50 mm was examined. This study also assessed the effect of using high strength concrete under the head region. The results showed that all specimens exhibited localized compressive failure of the concrete beneath the headed bars. Due to its increased number of sides, the hexagonal head performed better than the square head. When compared to a headed bar with a diameter of 12 mm, the ultimate local pressure of headed bars with diameters of 15, 17, 20, 25, 30, 40, and 50 mm dropped by 53, 60, 68, 76, 78, 87, and 90%, respectively. When the concrete cover-to-stud diameter increased from 5 to 30, there was a noticeable improvement in the final local pressure and the related slip under the rebar head. Overall performance of the bars improved with increase of the members number of the head. When high strength concrete was used under the head, the ultimate local pressure of headed bars increased by 29–152%. Many new formulas were proposed for estimating ultimate local pressure of headed bars.

* Corresponding authors: sabry_fayed@eng.kfs.edu.eg (S. Fayed).

## Introduction

Over the past few years, the construction industry has increasingly turned to exceptionally strong reinforcing bars^1–10^. These high-strength materials require longer embedment lengths due to their mechanical characteristics. Yet, space limitations often hinder their practical use. To resolve this, headed bars are employed to significantly reduce the required embedment length^11^. The use of headed reinforcement bars subjects the underlying concrete to a combination of compressive forces and bond stress. Numerous studies have focused on evaluating the anchorage behavior of such systems and the bond interaction between steel and concrete. Previous findings indicate that headed bars can effectively replace hooked reinforcements, providing enhanced ductility in anchorage performance^12,13^. According to Vella et al.^14,15^, headed bars offer significant benefits in the connection zones of precast concrete elements. Their research led to the development of a design method grounded in experimental data and numerical analysis. Maranan et al.^16^ reported that headed bars can enhance the tensile strength of GFRP bars by 49–77%, and that even anchorage using headed bars alone can result in a 45% improvement. Islam et al.^17^ examined several parameters affecting bond behavior and identified bar diameter, embedment depth, and concrete cover as critical factors.

Although headed bars enhance anchorage, they impose concentrated stress on a small concrete surface. Therefore, concrete bearing strength is essential for effectively transferring these localized compressive forces. This design consideration is critical in elements such as bridge pedestals, corbels, and anchors, which often experience high localized loads and uneven stress distribution, affecting overall stability. To better understand and accurately predict the concrete’s response under such localized stresses, several researchers have investigated its bearing strength through experimental and theoretical models. Bauschinger^18^ was among the first to evaluate the bearing strength of construction materials, introducing a cube-root equation based on limited tests on sandstone cubes. However, this approach has proven unreliable for predicting the behavior of concrete. Later, Shelson^19^ and Meyerhof^20^ observed that concrete failures under concentrated loads resemble those seen in triaxial compression tests. To describe this behavior, Au and Baird^21^ proposed an inverted pyramid failure model beneath the loading plate, attributing failure to induced tensile and bending stresses. Nonetheless, subsequent post-failure analyses have raised doubts about the accuracy of this model. In their study, Fayed et al.^22^ explored the relationship between concrete block size and its mechanical behavior, focusing on ultimate slip, bearing stiffness, and strength. The results showed a consistent reduction in both strength and stiffness with increasing block dimensions. Yehia et al.^23^ performed combined experimental and numerical analyses to investigate how sub-surface voids beneath the loaded plate influence bearing performance. Variations in hole diameter (6–18 mm) and depth (20–100 mm) revealed that the ultimate bearing capacity decreased by up to 35% when the hole-to-plate area ratio ranged from 1.4% to 40%.

Extremely high-strength reinforcing bars have become more and more popular in the building sector in recent years. Because of their mechanical properties, these high-strength materials need longer embedment lengths. However, its actual use is frequently hampered by space constraints. Headed bars are used to greatly shorten the necessary embedment length in order to address this. There is still a dearth of information in this field, particularly with relation to coding. Thus, the local pressure capacity of the concrete beneath the head of the reinforcing bars is the main focus of this study. The concrete cover, head form, head diameter, and depth of the high-strength concrete beneath the head are the primary objectives.

**Fig. 1:**
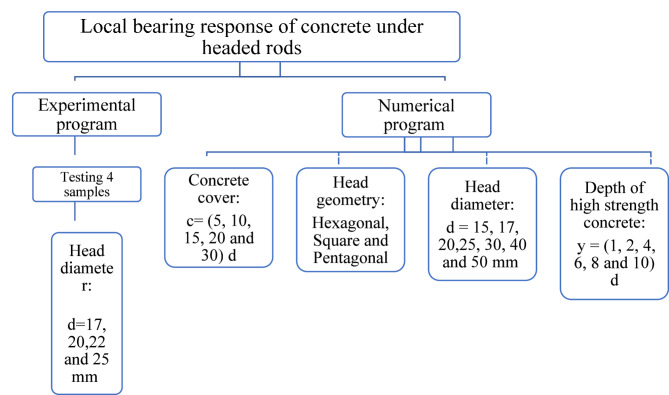
Flowchart of the current study.

In this study, behavior of headed reinforcement rods was numerically studied using 3D finite element method (FEM). First, an experimental program consists of four concrete specimens with variations only in head diameter was conducted. Second, FEM program consists of 22 concrete specimens was conducted. the effect of concrete cover thickness around the head was ranging from 85 mm to 510 mm. This variation resulted in a broad range of concrete cover-to-stud diameter ratios from 5 to 30. Additionally, studying the impact of head geometry (hexagonal, square, circular, and pentagonal) was performed. the effect of head diameter from 12 mm up to 50 mm was examined. This study also assessed the effect of using high strength concrete under the head region. In the current study, the headed bars are not embedded in the concrete to isolate the role of the bearing stresses below the rod head on the main variables in this study. However, if the reinforcing bars are buried in the concrete with the head, the result will be a result of two variables combined at same time. The experimental program tested herein can be summarized in Fig. 1.

## Experimental program

The experimental program consists of four concrete specimens sharing the same dimensions and bar head shape, with variations only in head diameter. The subsequent sections outline the methodology in detail, including specimen specifications, material properties, fabrication process, and testing and loading procedures.

### Materials

#### Normal concrete

The concrete specimens were prepared using a uniform normal concrete (NC) mix, as detailed in Table 1. The mixture comprised sand, crushed basalt dolomite (maximum particle size 15 mm), Grade 42.5 Portland cement, potable water, and Sikament-163 M as a superplasticizer. The mix was proportioned by weight at 1: 2.80 : 4.80 with a water-to-cement ratio of 0.5. In accordance with ECP 203/2018^24^, 150 mm cubes were cast to determine compressive strength, which averaged 20 MPa. Additionally, 150 × 300 mm cylinders were cast to evaluate tensile splitting strength, yielding a value of 1.8 MPa.

**Table 1:** Compositions of normal concrete (kg/m^3^).

Concrete mix type	Cement	Water	Fine aggregate	Coarse aggregate	Super plasticizer	water/cement (%)
NC	250	150	700	1200	2.5	0.50

#### Anchor bolt and head

**Fig. 2:**
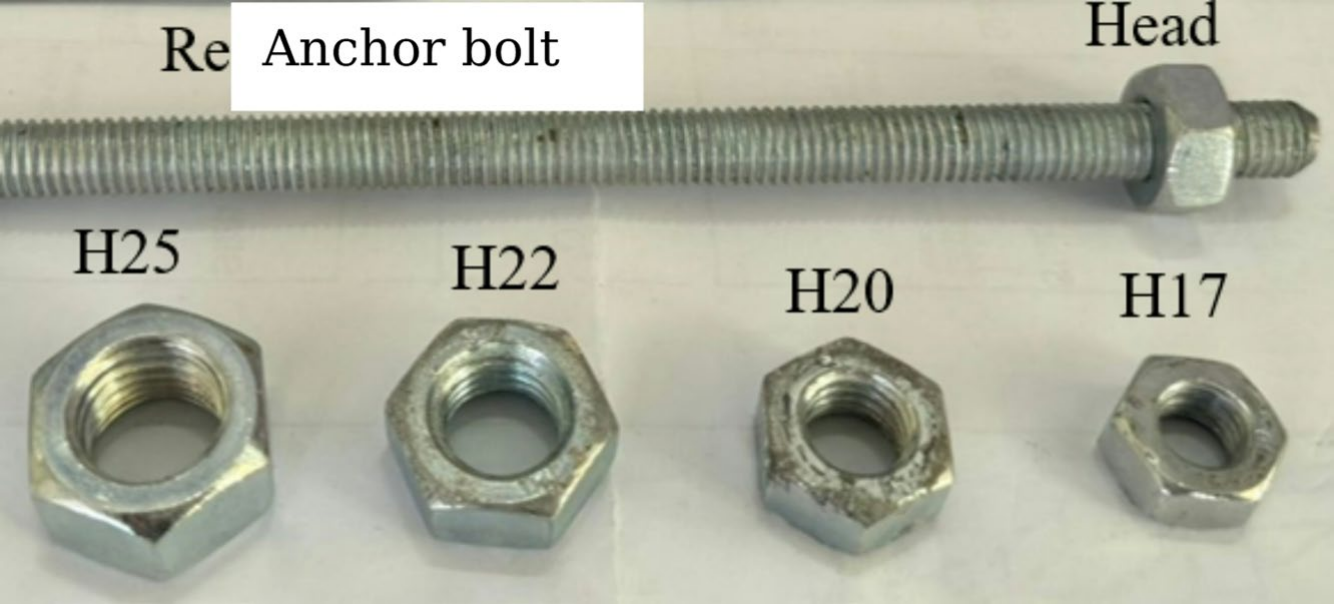
Anchor bolt and heads.

To simulate the head action of anchor bolt, mild steel nuts were affixed to the concrete surface, as shown in Fig. 2. The nuts were tested and reported by the manufacturer to have an elastic modulus of 202 GPa, with yield and ultimate strengths of 250 MPa and 350 MPa, respectively. The nuts featured hexagonal or round cross-sections, with four hexagonal sizes used: H17 (17 mm diameter, 5 mm height), H20 (20 mm, 6 mm), H22 (22 mm, 6.5 mm), and H25 (25 mm, 8 mm). A threaded steel rebar of 10 mm diameter, exhibiting 500 MPa yield and 700 MPa ultimate strength, was used to apply the compressive force on the nuts and simulate localized pressure on the concrete.

### Specimen’s details

**Fig. 3:**
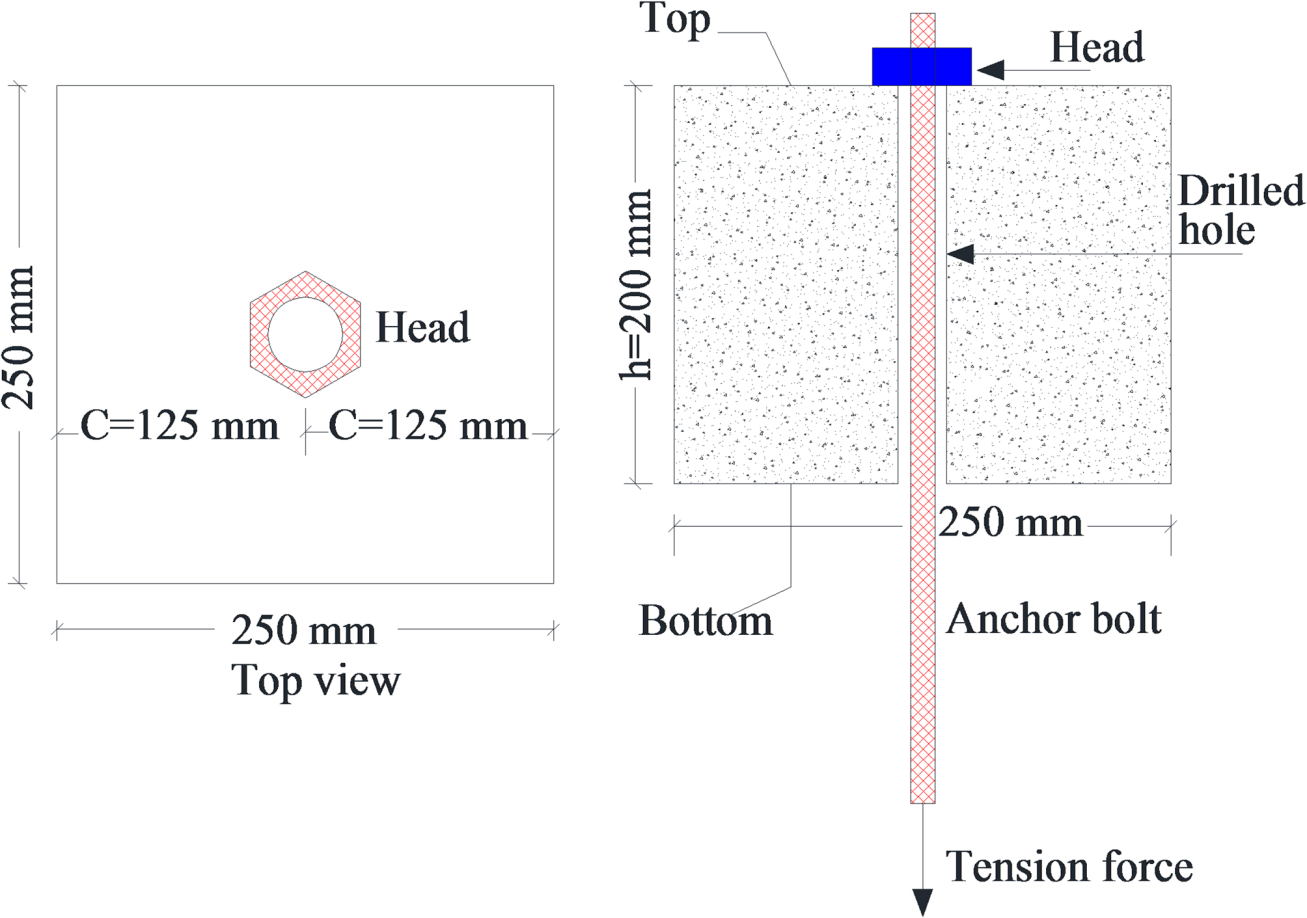
Details of tested specimens.

The experimental program involved four concrete specimens, each identified by the diameter and shape of the reinforcing bar head used. Each specimen was a block measuring 250 mm by 250 mm in cross-section and 200 mm in height (see Fig. 3; Table 2). All specimens featured hexagonal heads with diameters of 17 mm, 20 mm, 22 mm, and 25 mm, designated as H17, H20, H22, and H25, respectively. The gross area of the head increased with diameter, measuring 109.2 mm² for H17, 181.3 mm² for H20, 235.8 mm² for H22, and 327.4 mm² for H25. Each specimen maintained a constant concrete cover thickness of 125 mm to ensure consistent embedment conditions. The ratio of concrete cover to head diameter (c/d) decreased as head size increased, with values of 7.3 for H17, 6.2 for H20, 5.7 for H22, and 5.0 for H25, reflecting a reduced relative cover for larger heads. This variation in head size and corresponding parameters was designed to investigate the influence of head dimensions on the local stress distribution and anchorage behavior of the reinforced concrete specimens.

**Table 2:** Details of the concrete specimens.

Sample ID	Head diameter d (mm)	Head shape	Gross area of head A_h_ (mm^2^)	Concrete cover c (mm)	c/d
H17	17	Hexagonal	109.2	125	7.3
H20	20	Hexagonal	181.3	125	6.2
H22	22	Hexagonal	235.8	125	5.7
H25	25	Hexagonal	327.4	125	5

### Test setup

**Fig. 4:**
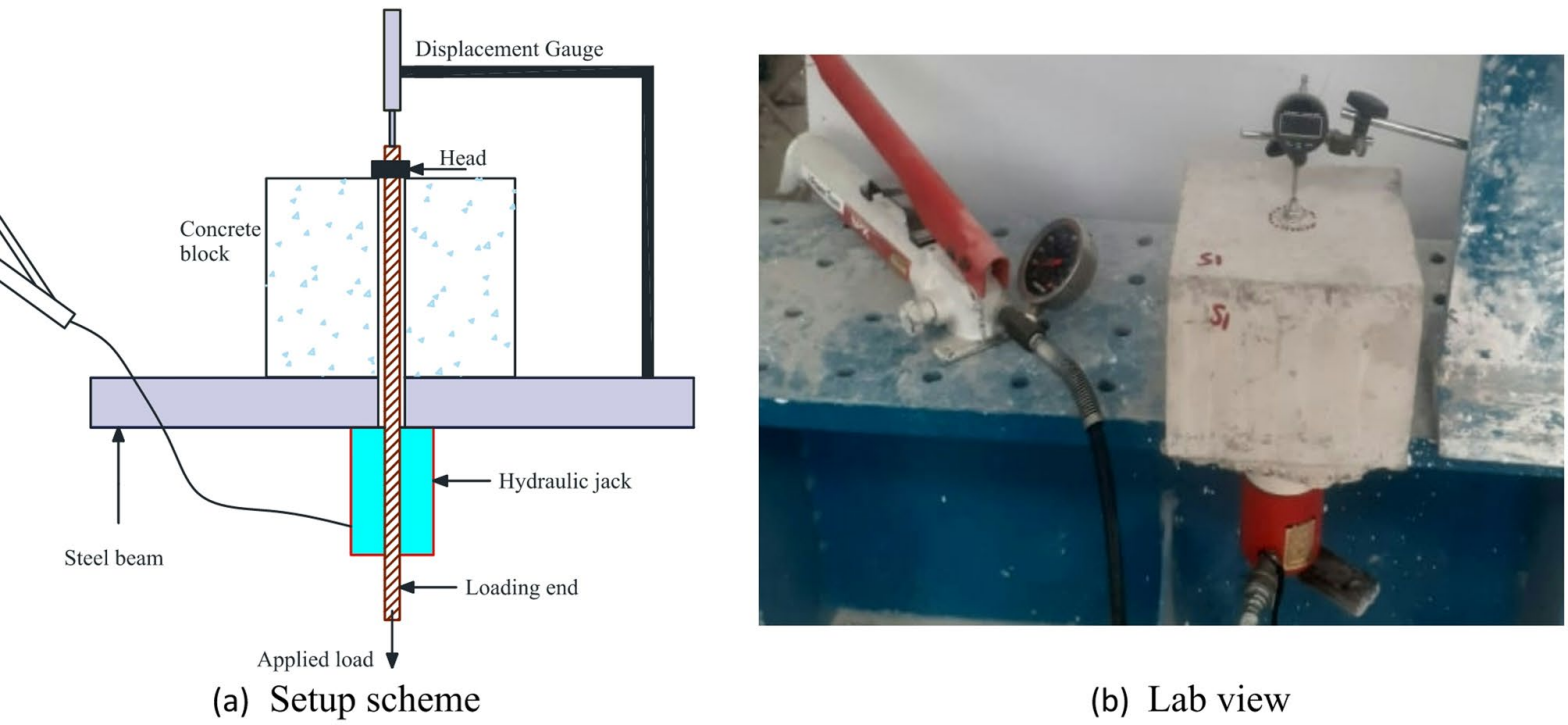
Test setup.

As depicted in Fig. 4, the test setup included a supporting steel beam, a centrally holed hydraulic jack, a displacement sensor, a headed anchor bolt, and a nut. Compression was applied to the headed bar using the hydraulic jack, which measured the force during loading. When tension force was applied on the anchor, the head will be going down within the concrete. The displacement sensor was vertically fixed to recorded the slip of the head (nut) within the concrete. Following the load test, the headed bar was removed, and the failure pattern beneath the head was photographed for further analysis.

## Results of experimental test and discussion

### Failure modes

**Fig. 5:**
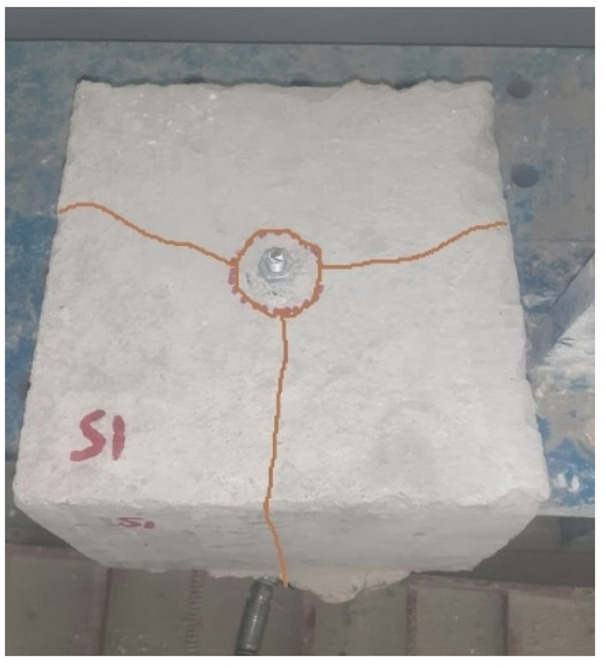
Typical failure mode of the tested specimen (H17).

Each specimen exhibited localized compressive failure of the concrete beneath the headed bars, resulting in substantial slip of the bars in the downward direction and gradual compaction of the adjacent concrete. The load is transferred from the rebar to the headed bar, which then distributes the force into the concrete mass. As illustrated in Fig. 5, a spreading zone of localized pressure forms beneath the headed bars, analogous to the anchor plates found in post-tensioning systems. The headed bar’s head progressively displaced downward under increasing load, and testing was ceased once complete slip of the head into the concrete was observed.

### General performance

**Fig. 6:**
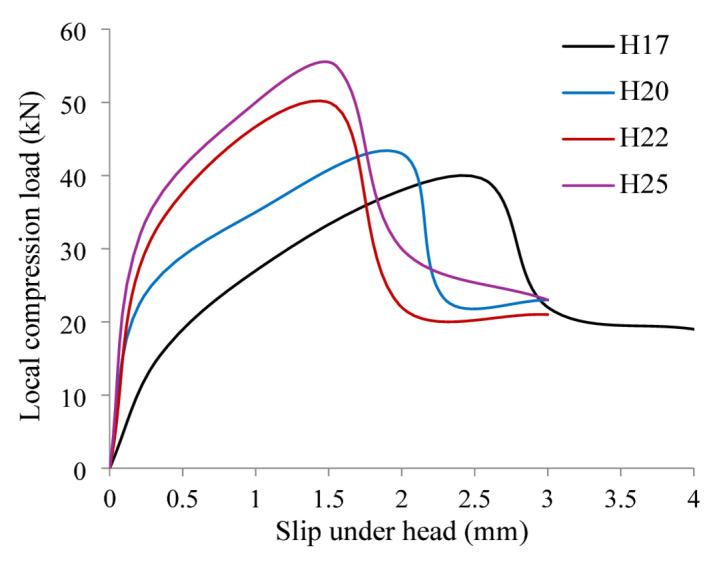
P-δ relationships of the tested specimens.

The applied local compression load versus head slip (P-δ) curves of all the tested specimens at various loading stages are shown in Fig. 6. Meanwhile, Table 3 summarizes the ultimate local bearing load ($\:{P}_{u}$) and the corresponding ultimate slip (δu) of the tested samples. In general, The P–δ response typically follows three sequential phases. The first phase, extending from initial loading to 50–60% of the peak load, is nearly linear, characterized by low slip (δ) and high load (P) gain. The second phase shows accelerated slip with decelerating load increase, ending at the maximum load. The third phase marks a nonlinear drop after peak load. This pattern was consistent across all sample groups. The findings demonstrated that the P–δ curve of sample H17, with the smallest head diameter (17 mm), exhibited the least enhancement, while sample H25, with the largest diameter (25 mm), showed the greatest improvement.

Additionally, as diameter (d) increased, $\:{P}_{u}$ also rose. According to Table 3, the maximum gain in $\:{P}_{u}$ was 42% at d = 25 mm. The measured $\:{P}_{u}$ values for H17, H20, H22, and H25 were 39, 43, 50, and 55 kN, respectively. Compared to H17, the increases in $\:{P}_{u}$ for H20, H22, and H25 were 11%, 28%, and 42%. This enhancement is attributed to the larger contact area under the head, which spreads the applied force over a wider concrete surface and delays localized failure. Head penetration (δu) is inversely related to head diameter (d). While all bars were placed centrally, the head diameter varied. With increasing d, a notable reduction in δu was observed. According to Table 3, δu decreased by 44% at d = 25 mm. Samples H17, H20, H22, and H25 recorded δu values of 2.5 mm, 2.1 mm, 1.5 mm, and 1.4 mm, respectively. These values reflect reductions of 16%, 40%, and 44% compared to H17. The decrease is attributed to the increased contact surface, which allows concrete to better resist penetration.

**Table 3:** Experimental results.

Sample	$\:{\boldsymbol{P}}_{\boldsymbol{u}}$ (kN)	Gain in $\:{\boldsymbol{P}}_{\boldsymbol{u}}$ (%)	slip δu (mm)	Decline in δu (%)
H17	39.1	0.00	2.5	0.00
H20	43.2	10.49	2.1	16.00
H22	50.1	28.13	1.5	40.00
H25	55.4	41.69	1.4	44.00

## FEM modelling

### Model development

**Fig. 7:**
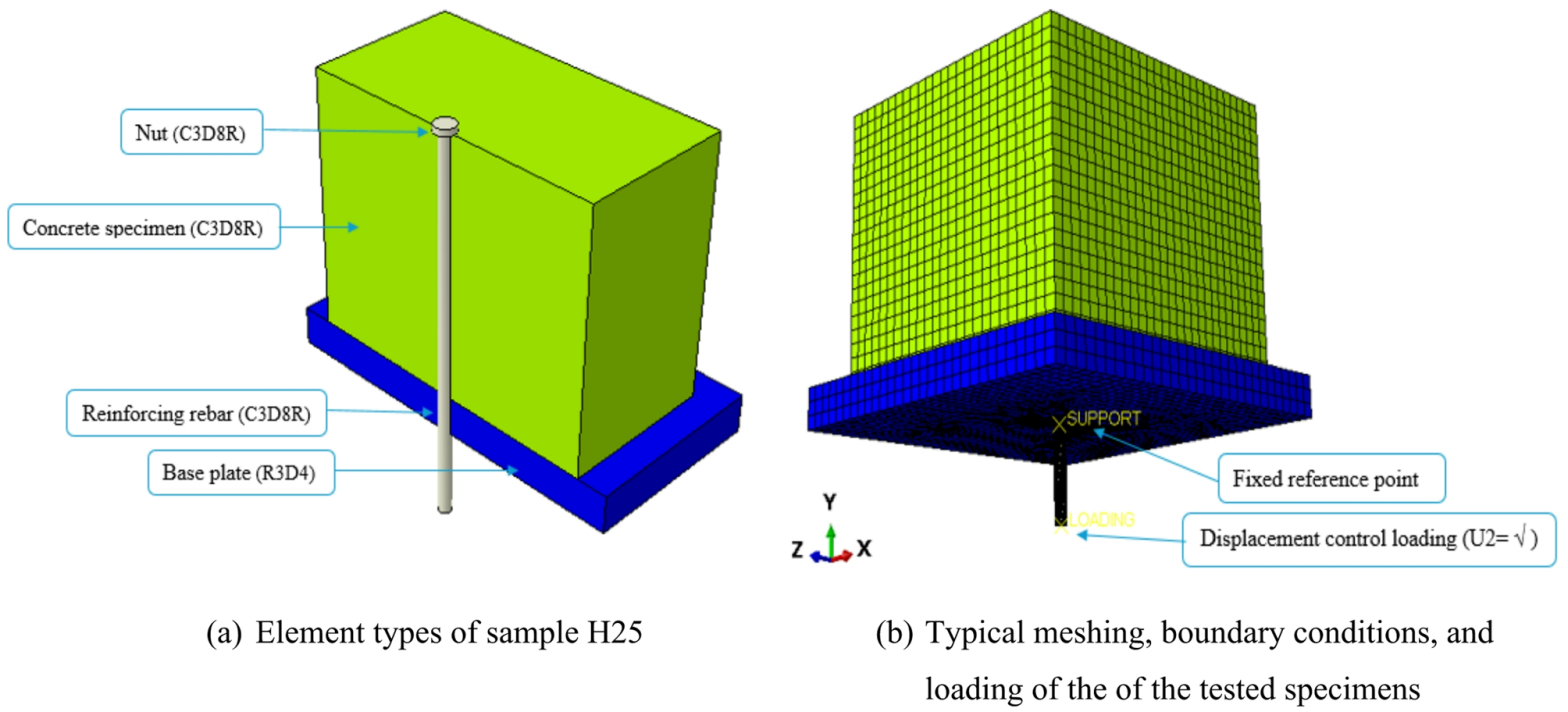
Simulation model of sample H25.

As illustrated in Fig. 7, a simulation model of sample H25 was constructed based on the experimental results. The concrete block, nut, and reinforcing bar were accurately modeled using 8-node reduced-integration brick elements (C3D8R). To capture the behavior of the rigid base plate, 4-node rigid surface elements (R3D4) were used, which are specifically designed to simulate contact with non-deformable surfaces (Fig. 7a).

Following the procedure outlined in^23^, the interaction between the concrete block and the base plate was modeled using surface-to-surface contact. Normal behavior employed a hard contact model to avoid penetration, and tangential behavior captured frictional effects, enabling the transfer of shear and normal forces across the interface. Friction was represented by the penalty method with a coefficient of 0.3, effectively simulating the steel–concrete interaction and enhancing simulation reliability. Additionally, a tie constraint was introduced between the nut and the concrete block to enforce complete connectivity, preventing separation or sliding and accurately reflecting the experimental observations.

To optimize the mesh configuration for the concrete block model, a mesh sensitivity study was undertaken. Of the sizes tested (5–20 mm), a 10 mm mesh achieved an effective compromise between computational time and solution accuracy, confirmed through comparison with experimental data. A finer mesh of 1 mm was applied to both the nut and reinforcing bar to capture local stress concentrations and detailed distribution, as illustrated in Fig. 7b. For boundary conditions, all degrees of freedom at the rigid base plate’s reference point were constrained to simulate a fixed support. The loading was introduced by incrementally displacing the reinforcing bar’s reference point, enabling realistic simulation of axial load transfer into the concrete block.

### Materials

#### Normal concrete

In this study, the Concrete Damage Plasticity (CDP) model was adopted to replicate the complex stress behavior of normal concrete (NC), including both compressive and tensile characteristics^25–35^. The compressive behavior was modeled using the Carreira and Chu stress–strain relationship^36^ (Eqs. 1–3), which is well-suited for NC. To ensure a realistic tensile response, a two-stage bilinear stress–strain model was calibrated from experimental data, forming a key part of the modeling framework and improving the accuracy of the numerical predictions.

1 $$\:{f}_{c}={f}_{c}^{{\prime\:}}\left[\frac{\beta\:\left(\frac{{\epsilon\:}_{c}}{{\epsilon\:}_{c0}}\right)}{\beta\:-1+{\left(\frac{{\epsilon\:}_{c}}{{\epsilon\:}_{c0}}\right)}^{\beta\:}}\right]$$

2 $$\:{f}_{t}=\left\{\begin{array}{c}{f}_{tu}\left[1.2\frac{{\epsilon\:}_{t}}{{\epsilon\:}_{t0}}-0.2{\left(\frac{{\epsilon\:}_{t}}{{\epsilon\:}_{t0}}\right)}^{6}\right]\:\:\:\:\:\:\:\:0\le\:{\epsilon\:}_{t}\le\:{\epsilon\:}_{t0}\\\:\:\:\:\\\:\:\:\\\:{f}_{tu}\left[\frac{\frac{{\epsilon\:}_{t}}{{\epsilon\:}_{t0}}}{1.25{\left(\frac{{\epsilon\:}_{t}}{{\epsilon\:}_{t0}}-1\right)}^{2}-\frac{{\epsilon\:}_{t}}{{\epsilon\:}_{t0}}}\right]\:\:\:\:\:\:\:\:{\epsilon\:}_{t0}\le\:{\epsilon\:}_{t}\end{array}\right.$$

Where *f*_*c*_ and *ε*_*c*_ denote the stress and strain of the concrete, respectively, while *f’*_*c*_ and *ε*_*co*_ represent the peak compressive stress and its corresponding strain. Additionally, the parameter *β* is determined using Eq. 3, which is derived from the stress–strain relationship.

3 $$\:\beta \: = \left( {\frac{{f_c^{\prime \,}}}{{32.4}}} \right) + 1.55\:$$

A sensitivity analysis was carried out to fine-tune the primary parameters of the Concrete Damage Plasticity (CDP) model, ensuring its suitability for simulating different concrete materials. The analysis highlighted the critical role of the dilatation angle (ψ), with NC best characterized by a value of 25°. To maintain a balance between accuracy and computational demand, additional model parameters were calibrated accordingly. As recommended by ABAQUS documentation, default values were adopted for the flow potential eccentricity (e) and the biaxial-to-uniaxial compressive strength ratio (*f*_*b0*_/*f*_*c0*_).

#### Steel reinforcement

To replicate the mechanical behavior of steel components, a bilinear elastic–plastic constitutive model with isotropic hardening was employed. The stress–strain response was defined using the two-phase approach proposed Han and Huo^37^. In the elastic phase, steel components followed a linear response characterized by the elastic modulus and Poisson’s ratio obtained from experimental results. Post-yield behavior was modeled with strain hardening, using a reduced modulus equal to 1% of the elastic value.

### Computational results

**Fig. 8:**
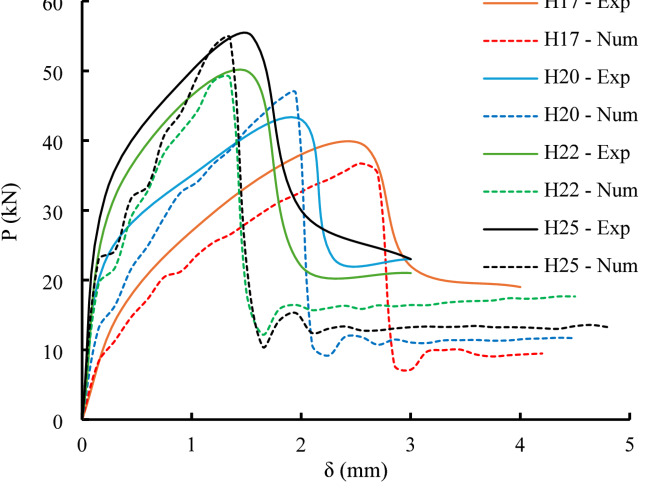
Comparison between experimental and numerical load – slip relationships.

**Fig. 9:**
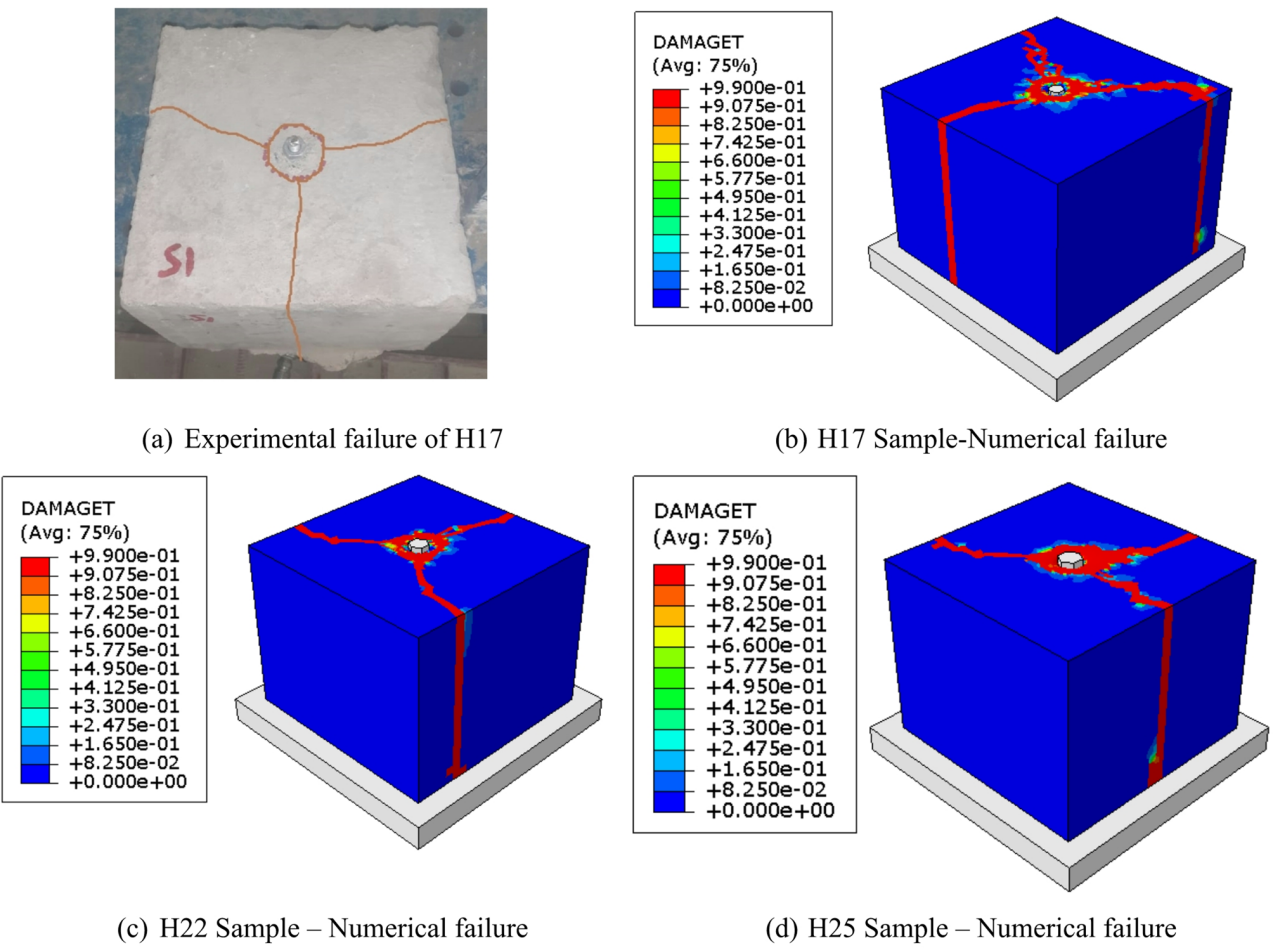
Comparison of experimental and numerical failure modes.

As indicated in Figs. 8 and 9, the numerical model showed a high degree of agreement with the experimental results, particularly in terms of load–slip performance, crack pattern development, and failure modes. The simulation effectively mirrored the complex sequence of structural behavior observed in testing. Table 4 offers further validation, reporting average µ values of 0.947 for load and 1.036 for slip, along with decreased standard deviations (0.042 and 0.024) and coefficients of variation (0.045 and 0.023). These metrics underscore the model’s predictive capability and support its application to broader structural analyses.

**Table 4:** A comparison of numerical versus experimental results.

Beam`s ID	$\:{\boldsymbol{P}}_{\boldsymbol{U}}$			$\:{\boldsymbol{\delta\:}}_{\boldsymbol{u}}$		
	$\:\boldsymbol{E}\boldsymbol{x}\boldsymbol{p}$	$\:\boldsymbol{F}\boldsymbol{E}$	$\:\boldsymbol{E}\boldsymbol{x}\boldsymbol{p}/\boldsymbol{F}\boldsymbol{E}$	$\:\boldsymbol{E}\boldsymbol{x}\boldsymbol{p}$	$\:\boldsymbol{F}\boldsymbol{E}$	$\:\boldsymbol{E}\boldsymbol{x}\boldsymbol{p}/\boldsymbol{F}\boldsymbol{E}$
H17	39.0	41.73	0.935	2.60	2.55	1.020
H20	43.0	46.44	0.926	2.0	1.87	1.070
H22	50.0	55.0	0.909	1.50	1.487	1.009
H25	55.0	54.0	1.019	1.55	1.48	1.047
µ			0.947			1.036
SD			0.042			0.024
CoV			0.045			0.023

## Parametric study

**Fig. 10:**
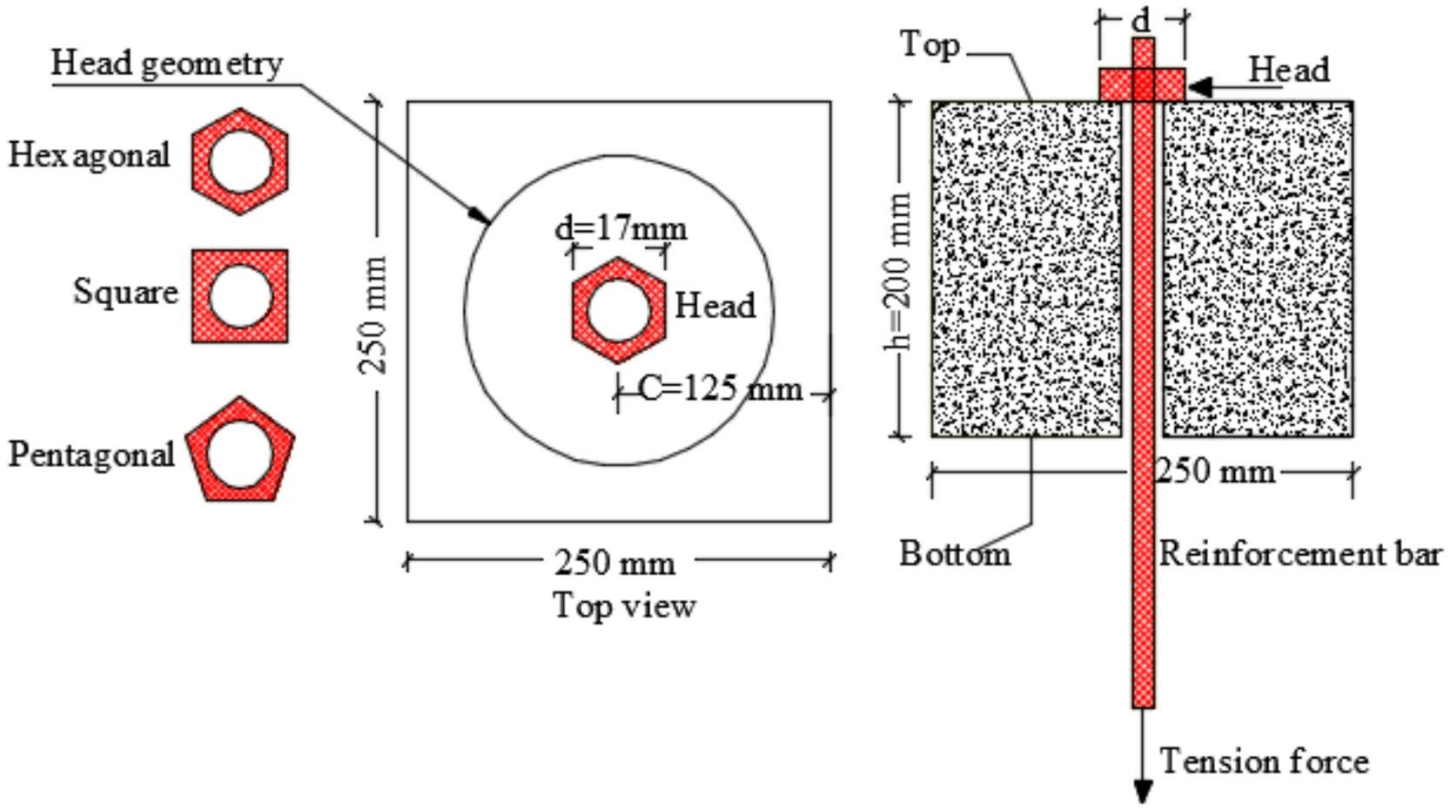
Key geometric parameters used in the parametric study (G2).

Based on the validated model, the current study conducted a comprehensive parametric study of concrete blocks with embedded headed bars. The specimens were divided into four distinct groups (G1 to G4), as shown in Table 5, each designed to isolate and examine the influence of key geometric and material parameters on their structural behavior (Fig. 10).

**Group G1** focused on the effect of concrete cover thickness. In this group, specimens shared a constant head diameter of 17 mm with a hexagonal head shape. The concrete block height was fixed at 200 mm, while the block length was varied significantly from 170 mm up to 1020 mm to achieve different concrete cover thicknesses ranging from 85 mm to 510 mm. This variation resulted in a broad range of concrete cover-to-stud diameter ratios (c/d) from 5 to 30. The primary aim was to examine how increasing the concrete cover influenced the load transfer capacity and failure modes of the concrete blocks.

**Group G2** was dedicated to studying the impact of head geometry on specimen performance. The specimens in this group had a fixed concrete block size with a cross-section of 250 mm by 250 mm and a height of 200 mm, along with a concrete cover thickness of 125 mm. All heads had a uniform diameter of 17 mm, but four different head shapes were tested: hexagonal, square, circular, and pentagonal. The gross cross-sectional area of the heads varied according to their geometry, from the smallest pentagonal head area of 93.3 mm² to the largest square head area of 210.5 mm², as listed in Table 5. Figure 10 shows details of this group.

**Group G3** examined the effect of head diameter size while maintaining a circular head shape. This group consisted of specimens with a constant block dimension and concrete cover thickness, but with head diameters ranging from 12 mm up to 50 mm. Correspondingly, the gross areas of the heads varied considerably, spanning from 34.56 mm² for the smallest diameter to 1885 mm² for the largest. This wide range enabled a detailed analysis of how increasing the head diameter—and thus the head area—affects the load capacity and interaction with the surrounding concrete.

**Group G4** assessed the effect of localized 50 MPa compressive strength at depth y below the top surface on the concrete block’s local compressive performance. All specimens in this group featured a 17 mm diameter hexagonal head and a constant concrete block dimension and cover thickness. However, the depth of concrete strength (y) influence was varied incrementally from d to 10 d (where d is the head diameter), spanning depths from 17 mm to 170 mm. This allowed investigation into how the depth over which concrete effectively contributes compressive resistance impacts the overall anchorage performance.

**Table 5:** Details of the specimens used in the parametric study (Parameters of numerical analysis).

Group	Specimens	Concrete block dimension		Head diameter	Gross area of head	Head shape	Concrete cover thickness	c/d	$\:{\boldsymbol{f}}_{\boldsymbol{c}}$depth	Parameter
		B (mm)	h (mm)	d (mm)	$\:{\boldsymbol{A}}_{\boldsymbol{h}}\:$(mm^2^)		c (mm)		y (mm)	
G1	H17- 5	170	200	17	109.2	Hexagonal	85	5	None	Study the effect of concrete cover thickness
	H17–10	340	200	17	109.2		170	10	None	
	H17–15	510	200	17	109.2		255	15	None	
	H17–20	680	200	17	109.2		340	20	None	
	H17–30	1020	200	17	109.2		510	30	None	
G2	H17	250	200	17	109.2	Hexagonal	125	7.3	None	Study the effect of head geometry
	S17	250	200	17	210.5	Square	125	7.3	None	
	P17	250	200	17	93.3	Pentagonal	125	7.3	None	
G3	C12	250	200	12	34.56	Circular	125	7.3	None	Study the effect of head diameter
	C15	250	200	15	98.2		125	7.3	None	
	C17	250	200	17	148.5		125	7.3	None	
	C20	250	200	20	235.62		125	7.3	None	
	C25	250	200	25	412.5		125	7.3	None	
	C30	250	200	30	628.3		125	7.3	None	
	C40	250	200	40	1178.1		125	7.3	None	
	C50	250	200	50	1885		125	7.3	None	
G4	H17- d	250	200	17	109.2	Hexagonal	125	7.3	17	Study the effect of concrete compressive strength depth
	H17- 2d	250	200	17	109.2		125	7.3	34	
	H17- 4d	250	200	17	109.2		125	7.3	68	
	H17–6d	250	200	17	109.2		125	7.3	102	
	H17–8d	250	200	17	109.2		125	7.3	136	
	H17–10d	250	200	17	109.2		125	7.3	170	

### Results and discussion of parametric study

#### Cracks and failure pattern

**Fig. 11:**
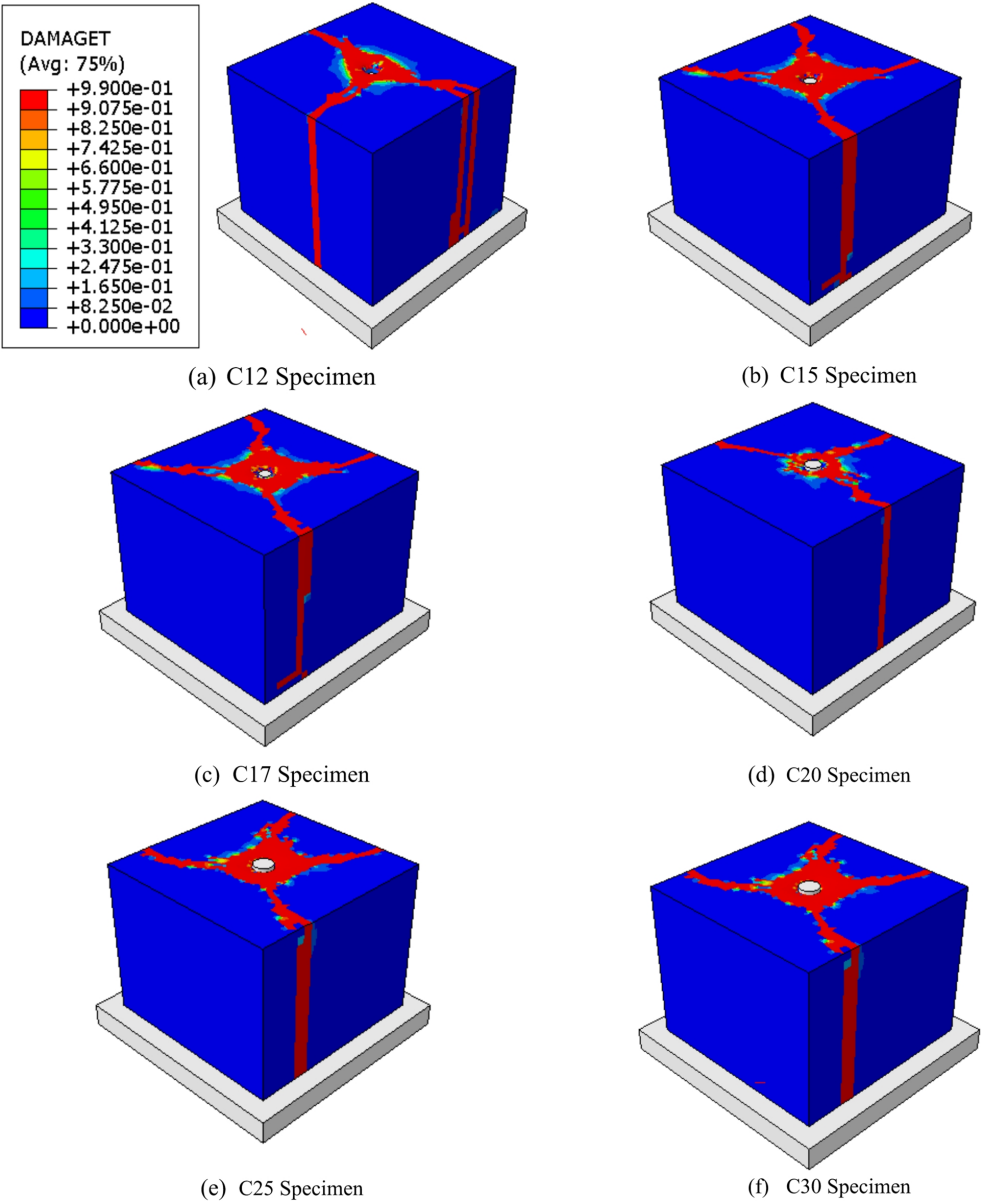
Failure mode of Group G3 specimens.

All specimens in groups G1 to G4 exhibited localized compressive failure of the concrete beneath the headed bars, resulting in significant downward slip of the bars and gradual compaction of the adjacent concrete, as previously discussed. The load is initially transferred from the rebar to the headed bar, which then distributes the force into the surrounding concrete block. As illustrated in Fig. 11, a spreading zone of localized bearing pressure develops beneath the headed bars.

Islam et al.^17^ investigated how various factors affected the bonding behavior of both headed-end and straight-end ribbed-surface bars embedded in the concrete. The effects of the bar diameter, the embedment length, end condition (with head and straight end) and the concrete cover on the bond strength were investigated experimentally using pullout test specimens. For both headed-end and straight-end bars, increasing the embedment length resulted in an increase in the capacity load.

But when it came to straight-end bars, these gains were more notable. There was no variation in the splitting force achieved for varying embedment depths in each example for headed-end bars because of distinct concrete splitting processes. This is because specimens with different embedment lengths and the same concrete cover had identical stress concentrations at the heads of the bars. The current study also shows a similarity in the collapse pattern below the bolt heads to the previous study^17^.

#### Ultimate local pressure

##### Effect of concrete cover thickness

**Fig. 12:**
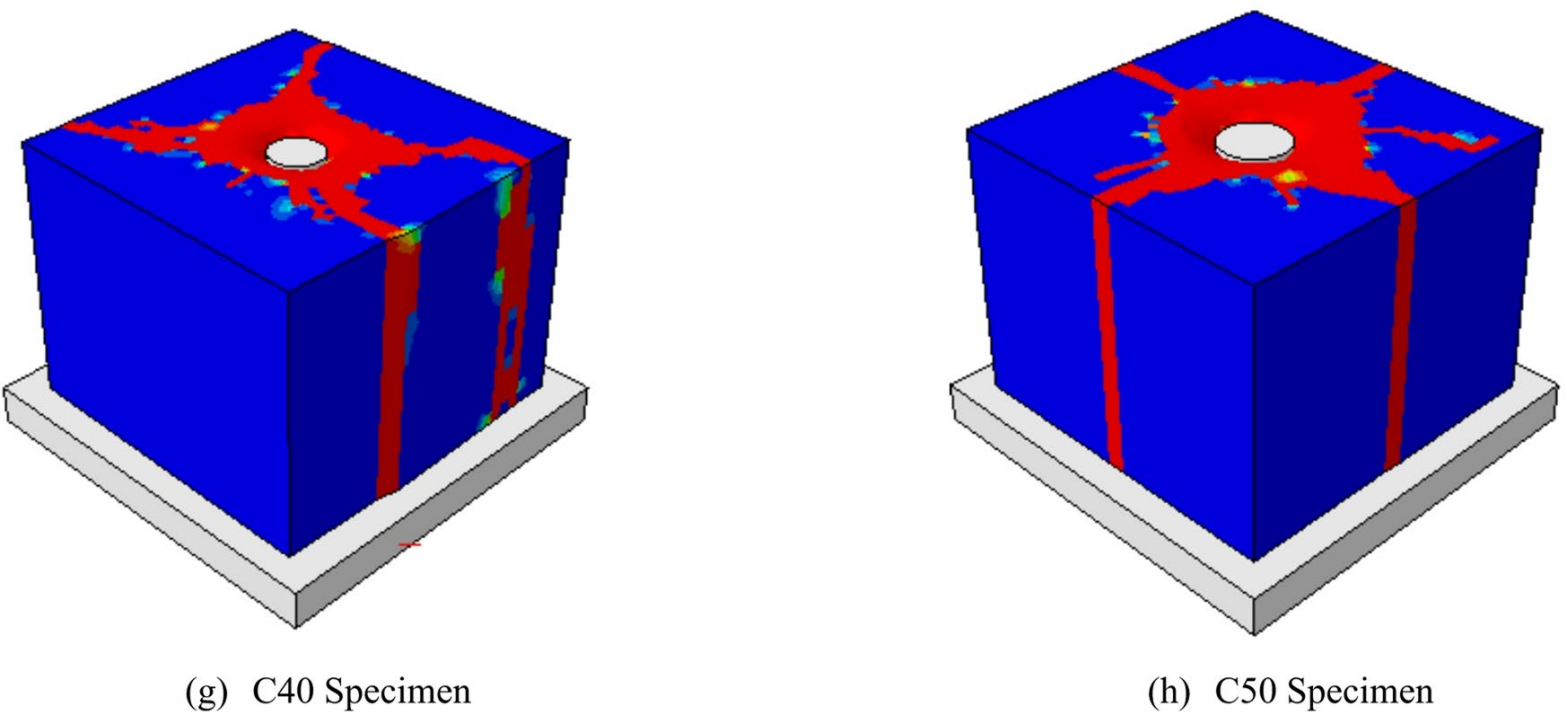
Effect of concrete cover thickness on the local pressure.

Figure 12 illustrates the effect of concrete cover thickness on the local pressure. The area of each bar head ($\:{A}_{h}$​) was obtained from Table 5. The ultimate local pressure ($\:{f}_{u}$​) for each specimen was calculated by dividing the ultimate load ($\:{P}_{h}$) by the corresponding head area ($\:{A}_{h}$). Relationships between local pressure and slip (*f*-δ) for different concrete cover thickness ratios were drawn in Fig. 12a. The *f*-δ curves improved more as c/d increased more. Values of the stress increased at the same level of slip when c/d increased more showing good impact. The fu of samples H17-5, H17-10, H17-15, H17-20 and H17-30 was 245, 313, 400, 541 and 768 MPa, respectively, while its corresponding slip was 1.3, 5.7, 14, 15 and 18 mm (Table 6). It was seen that both ultimate local pressure ($\:{f}_{u}$​) and its corresponding slip clearly improved with increase of the concrete cover-to-stud diameter ratios (c/d) from 5 to 30. Relationship between the $\:{f}_{u}$ and c/d was plotted in Fig. 12b. This relationship was linear. The reason for this noticeable improvement is the increased concrete enclosure below the bar head. The stronger the surrounding concrete, the better the concrete resistance below the bar head.

This study induced a new equation to estimate the fu of headed rods consedering effect of the concrete cover-to-stud diameter ratios (c/d) as below:

fu = 22.2 (c/d) + 100 (4).

**Table 6:** Outcomes of parametric study specimens.

Group	Sample	$\:{\boldsymbol{f}}_{\boldsymbol{u}}$ (MPa)	$\:{\boldsymbol{f}}_{\boldsymbol{u}}/{\boldsymbol{f}}_{\boldsymbol{u}0}\:\:$	δu (mm)	δu/δ_u0_
G1	H17- 5	251.9	1.00	1.35	1.00
	H17–10	288.6	1.15	5.55	4.11
	H17–15	403.6	1.60	13.95	10.33
	H17–20	548.6	2.18	14.25	10.56
	H17–30	779.6	3.09	17.55	13.00
G2	S17	210	1.00	1.50	1.00
	P17	300	1.43	1.65	1.10
	H17	340	1.62	2.55	1.70
G3	C12	663	1.00	4.05	1.00
	C15	314	0.47	2.10	0.52
	C17	264	0.40	2.10	0.52
	C20	211	0.32	1.65	0.41
	C25	158	0.24	1.35	0.33
	C30	142	0.21	1.35	0.33
	C40	83	0.13	0.75	0.19
	C50	61	0.09	0.30	0.07
G4	H17	340	1.00	2.55	1.00
	H17- d	440	1.29	1.05	0.41
	H17- 2d	720	2.12	1.50	0.59
	H17- 4d	790	2.32	1.65	0.65
	H17–6d	860	2.53	1.80	0.71
	H17–8d	860	2.53	1.80	0.71
	H17–10d	860	2.53	1.80	0.71

##### Effect of head geometry

**Fig. 13:**
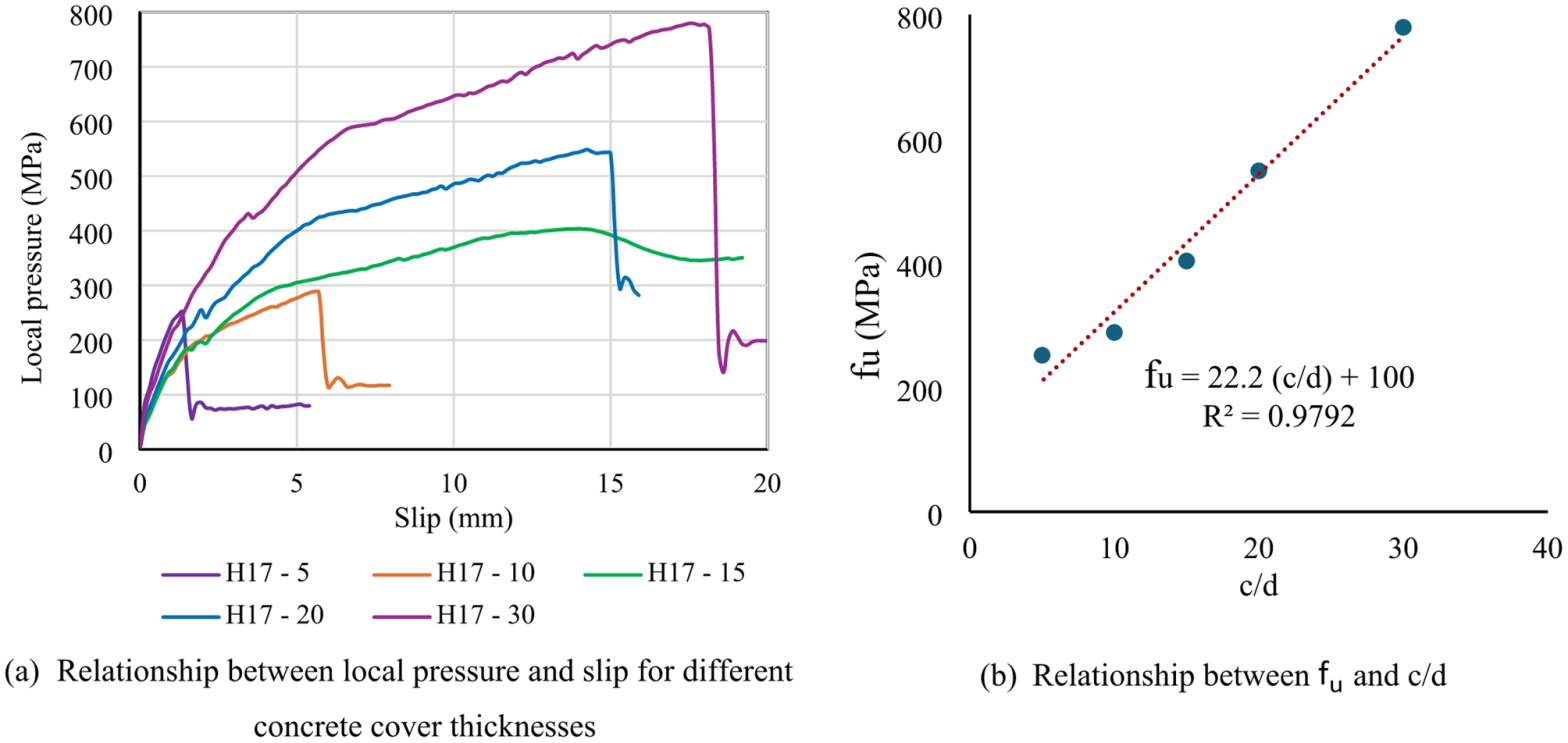
Effect of head geometry on the local pressure.

In group G2, Fig. 13 shows effect of head geometry on the local pressure of the samples S17, P17 and H17. All heads of these samples had a same outside diameter of 17 mm, but three different head shapes were tested: hexagonal in sample H17, square in sample S17, and pentagonal in sample P17. Sides number of the head were 4, 5 and 6 in S17, P17 and H17, respectively. The gross cross-sectional area of the heads was 210, 93 and 109 mm^2^ in S17, P17 and H17, respectively. The curves *f*-δ of this group were drawn in Fig. 13a. The *f*-δ curves improved more as sides number of the head increased more. Values of the stress increased at the same level of slip when sides number of the head increased more showing good impact. The fu of samples S17, P17 and H17 was 207, 300, and 322 MPa, respectively, while its corresponding slip was 1.5, 2 and 2.5 mm. It was seen that both ultimate local pressure ($\:{f}_{u}$​) and its corresponding slip clearly improved with increase of the members number of the head from 4 to 6 (Table 6).

It was showed that as the number of head edges increased, the $\:{f}_{u}$ clearly improved. The reason for this noticeable improvement is the intersection of the sides together creates corners, which causes stress concentration at these corners. The fewer the vertex edges, the fewer the corners. The fewer the corners, the more concentrated the load is at a single corner, accelerating collapse. For this reason, the lowest bearing capacity of all specimens is achieved by the square-vertex specimen, which has only four sides. Conversely, the highest bearing capacity is achieved by the hexagonal-vertex specimen, which has six sides. Therefore, the more vertex edges, the better the behavior.

##### Effect of head diameter

Group G3 investigated the impact of head diameter size while keeping a circular head shape. This group included specimens with the same block dimension and concrete cover thickness, but with head diameters varying from 12 mm (C12) to 50 mm (C50). The gross areas of the heads differed significantly, ranging from 34.56 mm² in sample C12 to 1885 mm² in C15.

**Fig. 14:**
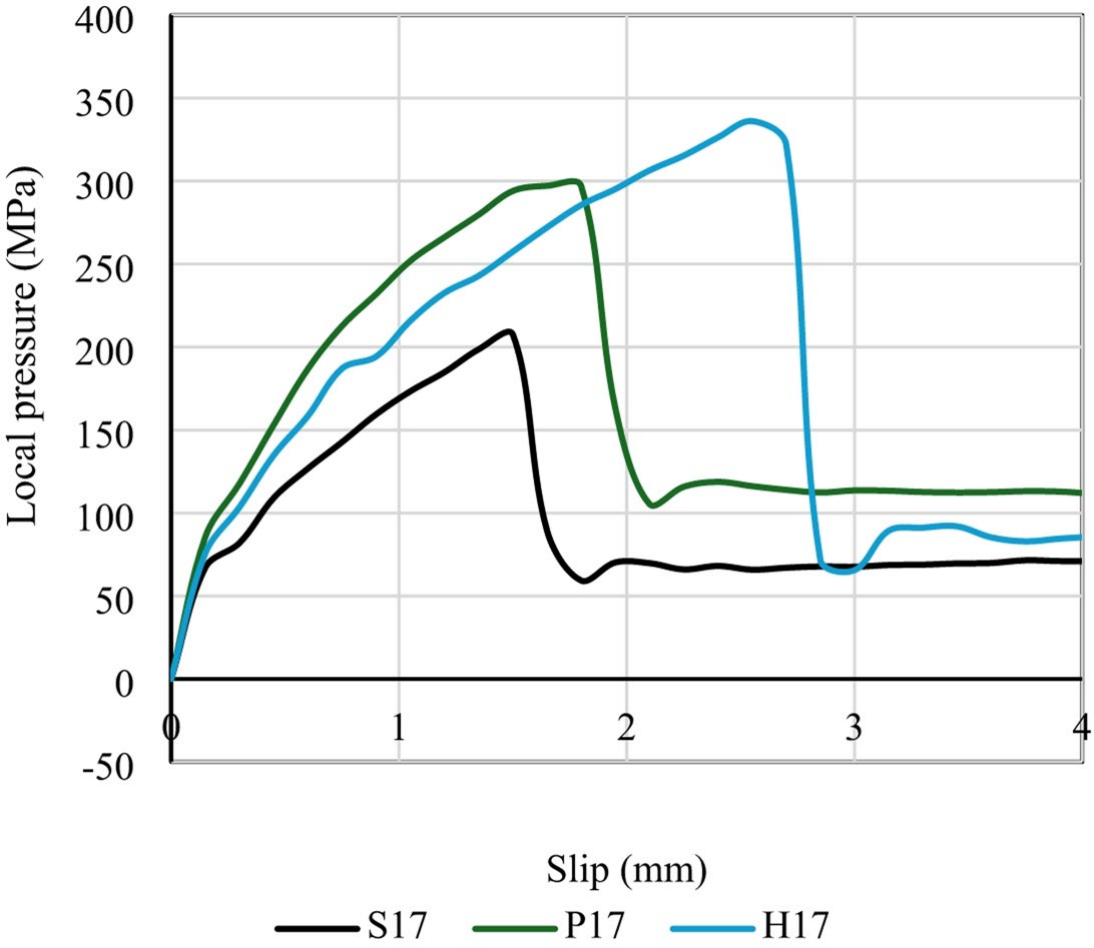
Effect of head diameter on the local pressure. (**a**) Relationship between local pressure and slip for different head diameters, (**b**) Relationship between fu and head diameter, (D), (**c**) Relationship between fu and head area, (Ah).

Figure 14 depicts the influence of head diameter on the local pressure of the following samples: C12, C15, C17, C20, C25, C30, C40, and C50. Figure 14a depicts the f-δ curves for this group. The f-δ curves improved more as the head diameter decreased. Local stress values rose at the same amount of slip as the diameter of the head decreased. The fu values for samples C12, C15, C17, C20, C25, C30, C40, and C50 were 663, 314, 264, 211, 158, 142, 83, and 61 MPa, respectively. The fu produced 663 MPa with a head diameter of 12 mm in sample C12. In contrast, when the head diameter approached 50 mm in sample C50, the fu decreased to 61 MPa. In other words, as compared to sample C12, the fu of samples C15, C17, C20, C25, C30, C40, and C50 fell by 53, 60, 68, 76, 78, 87, and 90%, respectively (Table 6). Figure 14b depicts the fu values versus head area (Ah). It was discovered that values of local stress fell significantly as the area of the head rose.

Relationship between the $\:{f}_{u}$ and head diameter (D) was plotted in Fig. 13b while Relationship between $\:{f}_{u}$ and head area (A_h_) was plotted in Fig. 14c. also, new formulas were induced in this study. It is clear that the two relationships are similar. As the diameter or area of ​​the head increases, the stress drops rapidly from 663 MPa to 263 MPa in a small horizontal distance. When the diameter of the nail increases from 12 mm to 17 mm, the stress drops rapidly from 663 MPa to 263 MPa. Then, as the head diameter increases above 17 mm, the severity of the stress drop decreases. As the head diameter increases above 30 mm, the stress stabilizes as it continues horizontally.

This study induced a new equations to estimate the fu of headed rods consedering effect of diameter (D) and area (Ah) of the head as below:

4 $$fu = 21863D-1.514$$

5 $$fu = 4685.6(Ah)-0.567$$

##### Effect of localized 50 MPa compressive strength at different depths

Group G4 evaluated how the local compressive ability of the concrete block was affected by the localized 50 MPa compressive strength at depth y below the rebar head. Each specimen in this group has a hexagonal head with a diameter of 17 mm. High strength concrete (50 MPa) was used to replace the concrete block’s top portion’s compressive strength with depth y. From d to 10 d (where d is the head diameter), the depth of concrete strength (y) influence was gradually changed, covering depths ranging from 17 mm to 170 mm. The depth y was 0, 17, 34, 68, 102, 136 and 170 mm in samples H17, H17- d, H17- 2 d, H17-4d, H17- 6 d, H17- 8 d, and H17- 10 d, respectively.

**Fig. 15:**
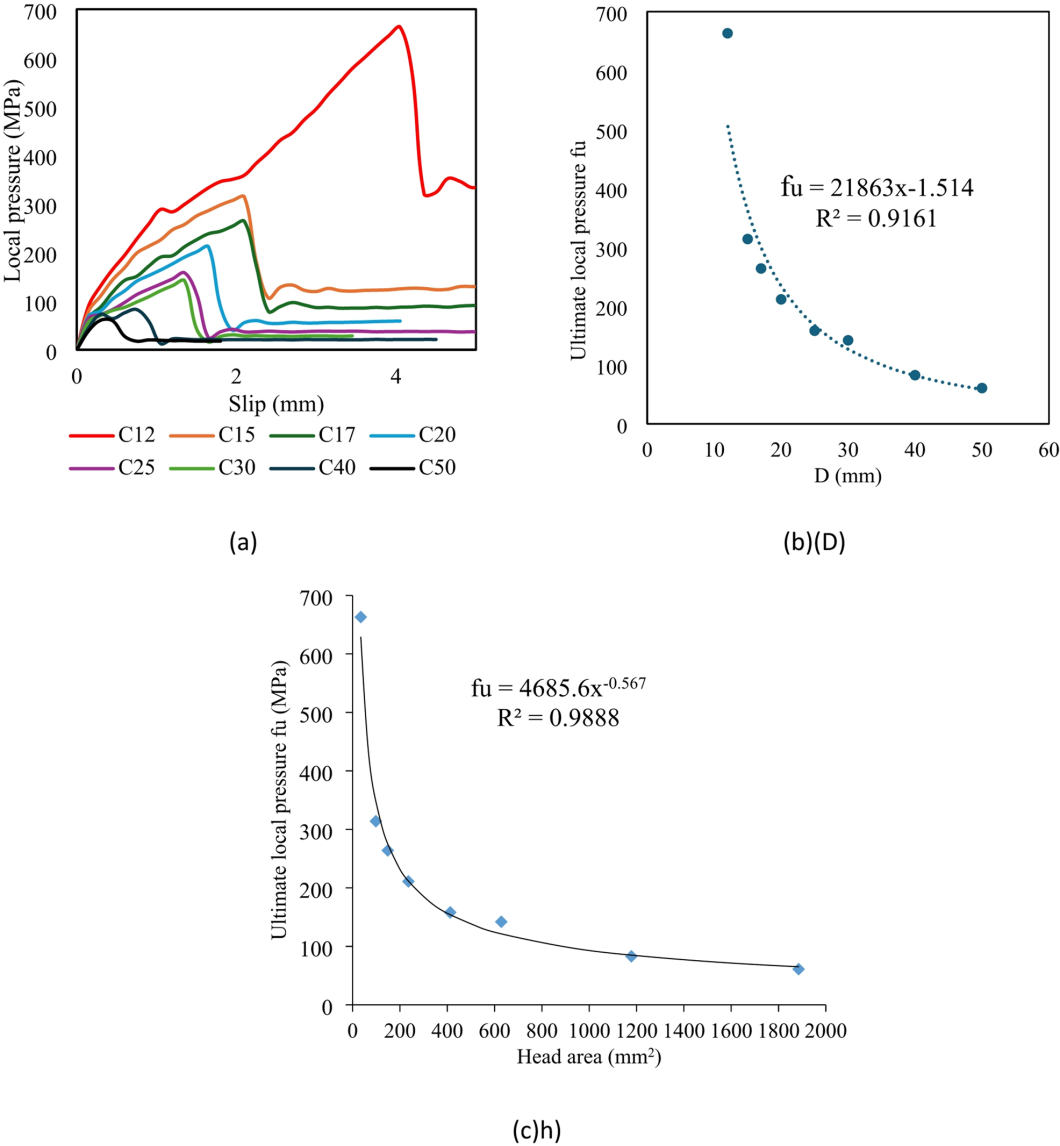

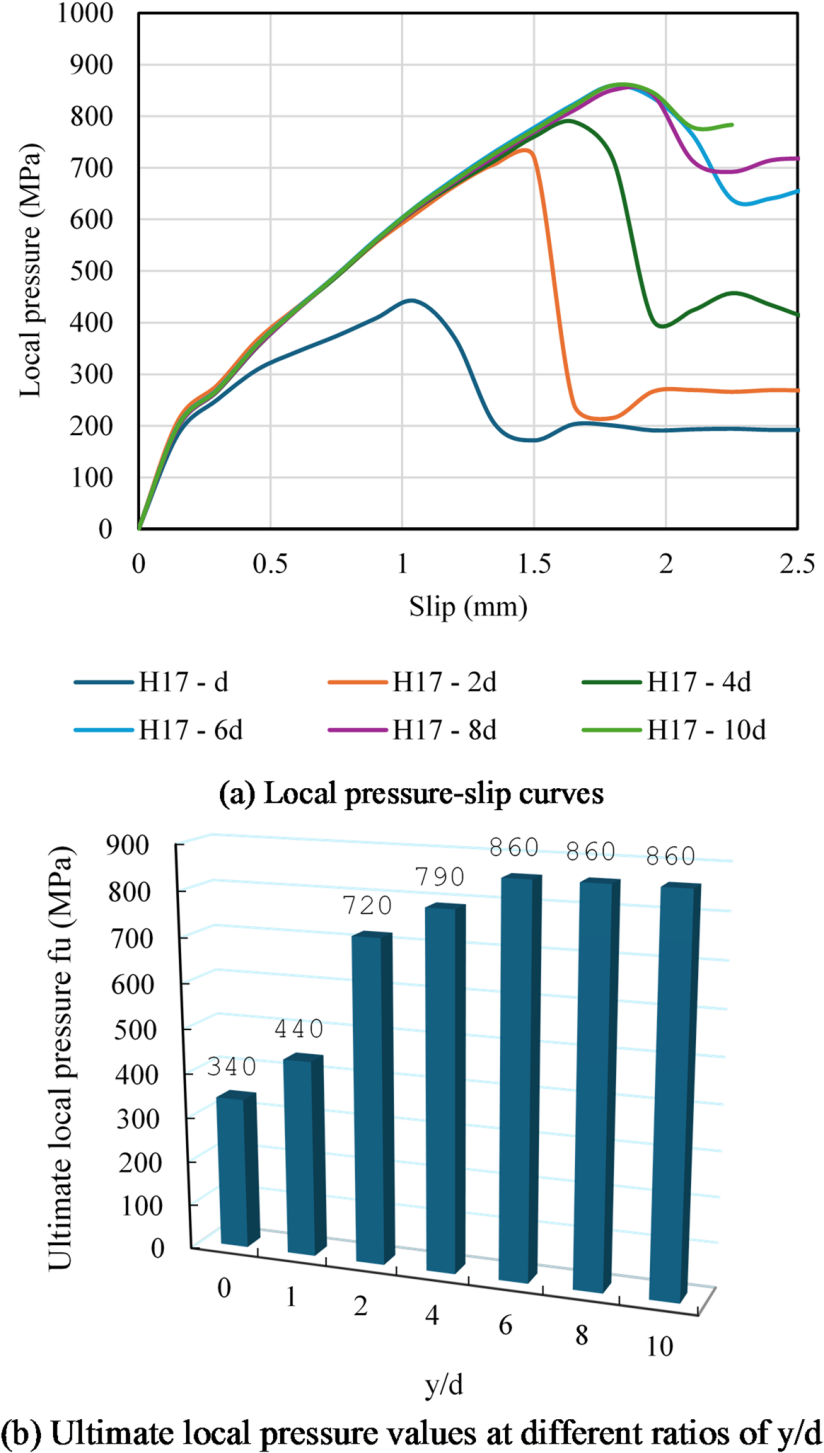

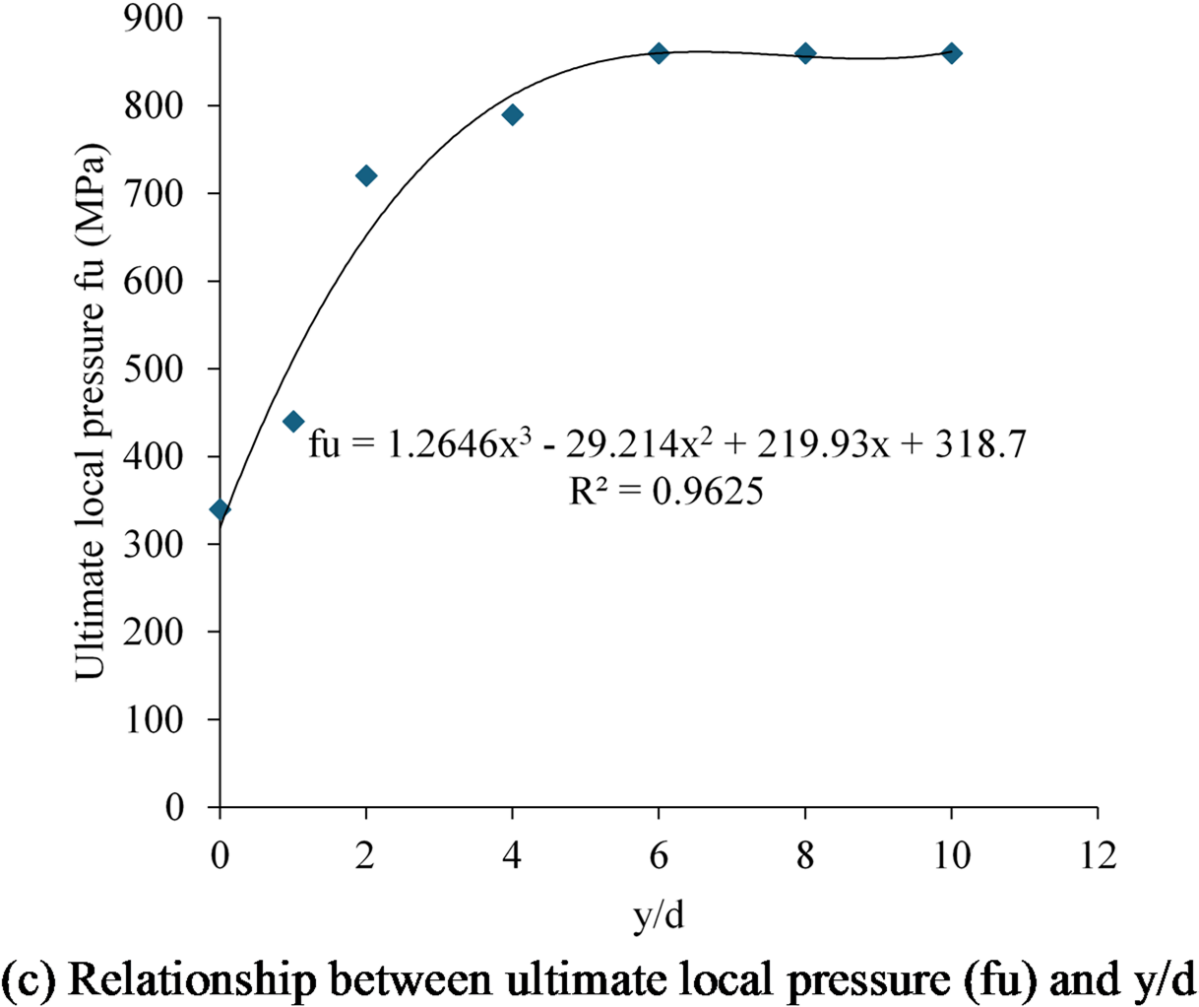
Effect of localized 50 MPa compressive strength at depth y on the local pressure. (**a**) Local pressure-slip curves, (**b**) Ultimate local pressure values at different ratios of y/d, (**c**) Relationship between ultimate local pressure, (fu) and y/d.

Figure 15 depicts the influence of high strength concrete (50 MPa) under the head on the local pressure of the following samples: H17, H17- d, H17- 2 d, H17-4d, H17- 6 d, H17- 8 d, and H17- 10d. Figure 15a depicts the f-δ curves for this group. The f-δ curves improved more as the depth y increased. Local stress values rose at the same amount of slip as y increased. The fu values for samples H17, H17-d, H17-2d, H17-4d, H17-6d, H17-8d, and H17-10d were 340, 440, 720, 790, 860, 860, 860, and 860 MPa, respectively (Table 6). Compared to sample H17, the fu improved by 29, 111, 132, 152, 152, 152% in samples H17-d, H17-2d, H17-4d, H17-6d, H17-8d, and H17-10d. Also, increasing ratio y/d from 0 to 1, 2, 4, 6, 8 and 10 enhanced the fu by 29, 111, 132, 152, 152, 152% (see Fig. 15b). Relationship between $\:{f}_{u}$ and ratio y/d was plotted in Fig. 15c. It was discovered that values of local stress increased significantly as the ratio y/d rose up to 4 but the stress fu stabilizes as it continues horizontally after the y/d exceeded 4.

This study induced a new equation to estimate the fu of headed rods consedering effect of y/d as below:

Where d is the head diameter and y is the depth of high strength concrete under the head.

## Comparison with previous studies

In the current study, the results showed that using a bar head significantly improved the concrete’s strength in the bearing. This has also been studied in previous research^17^ using rods with a head compared to rods without a head. The current results must be compared with the results of previous studies; therefore, this section will do that. The findings of previous study^17^ proved that when headed-end bars were used instead of straight-end bars, the ultimate load and bond stress increased significantly for all embedment lengths and concrete cover values. In the case of headed-end bars without extra embedment lengths, the heads’ embedment lengths were 75 mm for bars with a diameter of 12 mm and 100 mm for bars with a diameter of 16 mm. In comparison to straight-end bars with embedment lengths of 6 times bar diameter for the 12 and 16 mm bar diameters, respectively, these examples displayed increased bond stresses of around 168% and 105%. In summary, compared to straight-end bar with the same embedment length as the head length, the produced bar heads’ design results in increased bond stress.

Furthermore, when the reinforcement is secured using headed bars, the concrete underneath the headed bars is affected by both the bond stress and the pressure force of the headed bars, according to other earlier research^11^. Thirty concrete pull-out specimens under various conditions were used in an experimental investigation. The findings demonstrated the production of a wedge under the local pressure region and the formation of a structure resembling a shell structure as a result of the pull rob’s action beneath the wedge. Additionally, as the concrete’s strength and loaded area increased, so did the concrete’s local bearing capacity. The present study’s findings are comparable to these.

A direct pullout test was used in a prior work^16^ to examine the impact of the anchor head on the pullout behavior of the sand-coated glass-fiber-reinforced polymer bars placed in the geopolymer concrete. Consideration was given to both straight and headed bars with varying nominal diameters and embedment lengths for headed bars. The findings demonstrated that adding an anchor head is a successful way to increase the bars’ ability to anchor in geopolymer concrete. The anchoring of the bars was enhanced by the anchor heads by up to 49–77%. Additionally, around 45% of the bars’ nominal tensile strength was developed as a result of the anchor head’s mechanical bearing resistance alone.

## Conclusion

Headed bars are used to drastically cut down on the amount of embedment length needed for high-strength reinforcement rebars, which require lengthier embedment lengths in the concrete. However, under the head, they apply focused stress on a little concrete surface. This study used 3D finite element method (FEM) to quantitatively investigate the behavior of headed reinforcing rods. First, a program of experiments was carried out using four concrete specimens that differed only in the head diameter. Second, a procedure using 22 concrete examples for FEM was carried out. The thickness of the concrete cover surrounding the head varied between 85 and 510 mm. This variation produced concrete cover-to-stud diameter ratios ranging widely, from 5 to 30. The effects of head geometry (hexagonal, square, circular, and pentagonal) were also investigated. The impact of varying the head diameter from 12 mm to 50 mm was investigated. The impact of employing high-strength concrete beneath the head region was also evaluated in this investigation. The failures, local pressure-slip curves, ultimate local stress and its corresponding slip of the headed bars were analyzed. The results showed the following:

1. The concrete beneath the headed bars showed localized compression failure in every specimen.
2. As the concrete cover-to-stud diameter ratios (c/d) increased from 5 to 30, there was a noticeable improvement in the final local pressure and the related slip.
3. Both ultimate local pressure and its corresponding slip clearly improved with increase of the members number of the head from 4 to 6. The hexagonal head was better than square head.
4. The ultimate local pressure of headed bars with diameters of 15, 17, 20, 25, 30, 40, and 50 mm decreased by 53, 60, 68, 76, 78, 87, and 90%, respectively, in comparison to a headed bar with a diameter of 12 mm. The local stress roughly stabilizes when the head diameter rises above 30 mm.
5. Depending on the difference in the replacement depth of the concrete beneath the head, the ultimate local pressure of headed bars increased by 29–152% when high strength concrete was used under the head.
6. Many new formulas were proposed for estimating ultimate local pressure of headed bars considering effect of high strength concrete depth, diameter and area of the head, and the concrete cover thickness.

## Data Availability

The datasets used and/or analysed during the current study available from the corresponding author on reasonable request.

## Declarations

### Practice application

This study shows that headed reinforcement anchors, especially high strength material bars, are a very practical and efficient way to restrict the length of the reinforcement bars inside the concrete. The results enable the safe and effective integration of critical service systems in contemporary structures without sacrificing structural safety and performance by offering structural engineers useful, optimal design recommendations.
